# “I don’t think service changed, I think people changed”: Palliative care delivery in Aotearoa/New Zealand after COVID-19

**DOI:** 10.1177/26323524251343095

**Published:** 2025-06-24

**Authors:** Rosemary Frey, Tess Moeke-Maxwell, Jackie Robinson, Deborah Raphael, Lisa Williams, Erica Munro, Jenny Thurston, Merryn Gott

**Affiliations:** 1 School of Nursing, Faculty of Medical and Health Sciences, University of Auckland, New Zealand; 2 Mercy Hospice, Auckland, New Zealand

**Keywords:** COVID-19, palliative care, self-determination, New Zealand

## Abstract

**Background:**

As a result of COVID-19 restrictions, palliative care services in New Zealand and across the world needed to adapt rapidly and creatively to find new ways of working, revising, and establishing new policies and practices. This article reports the findings of phase I of an 18-month study examining changes in hospice care delivery in Aotearoa/New Zealand in the wake of COVID-19.

**Objective:**

This study aimed to explore the motivations underpinning adaptations and innovations in the delivery of palliative care in the wake of COVID-19.

**Design:**

Transdisciplinary Action Research and Partnership approaches were utilised.

**Method:**

A thematic analysis was conducted of open-ended telephone, video, and in-person questionnaire data collected from January to August 2024 from a diverse group of stakeholders. Our findings explored the motivations for health professional practice changes in the realms of competence, relationships, and autonomy.

**Findings:**

Changes in behaviour involved streamlining and adapting policies and services, using technology to facilitate communication, building collaborative connections, and activating health professionals, families, and Indigenous empowerment.

**Conclusion:**

Palliative care services needed to adapt rapidly and creatively to the threats posed by COVID-19. The threats posed were a motivator to shift thinking about palliative care delivery regarding services, relationships, and empowerment. This shift in thinking led to changes in ways of working, revising, and establishing new policies and practices. The driving force underpinning the changes and innovations is the desire to provide patient-centred care. Indeed, the findings build a case for patient-centred, sustainable, and effective innovation. From the perspective of health professionals, the findings may inform strategies to sustain new practices in delivering quality palliative care. Additionally, they may also provide insights into possible methods to grow individual and community capacity to face future pandemics.

## Plain language summary

Palliative care delivery in Aotearoa/New Zealand after COVID-19

*Why was the study done?* The study was done to understand why some changes made to palliative care services during the COVID-19 pandemic were kept even after the pandemic ended. Health services had to adapt quickly, and the researchers wanted to explore why these changes continued.

*What did the researchers do?* The researchers interviewed 37 professionals, including health workers and managers in hospice organizations in Aotearoa New Zealand, and worked with a Māori researcher to include the views of Māori communities.

*What did the researchers find?* The study found that changes included improving services, using technology for better communication, working together more, and promoting independence for families and Indigenous people. The reasons these changes stuck were linked to factors like feeling more skilled, building better relationships, and having more control over work.

*What do the findings mean?* The findings indicate the importance of patient-centred care. They help us understand what motivates health professionals to keep using new ways of providing palliative care and how to support them in maintaining these changes.

The World Health Organisation (WHO) explains that “integrated care is often contraposed to fragmented and episodic care, and it is used synonymously to terms like coordinated care and seamless care, among others.”^1^ Yet, such integrated care during the COVID-19 pandemic became challenging both during the pandemic and beyond. Indeed, the COVID-19 pandemic outbreak severely affected healthcare organisations worldwide^2^ particularly because people with chronic life-limiting illnesses were considered a high-risk group in terms of COVID-19-related morbidity and mortality.^2^ During the pandemic of COVID-19, palliative care patients were considered one high-risk group in the morbidity and mortality of COVID-19.^3^ Restrictions to hospice visiting and limitations in bereavement support increased psychosocial distress for patients and families.^4,5^ Community palliative care delivery was scaled back, leading to gaps in continuity of care and failure to address the physical, psychological, social, and spiritual needs of patients and caregivers, at a time when they most required it.^6^ The “Lancet Commission on the Value of Death” proposed the need for far-reaching changes to the heart of hospice service provision to meet the challenges of a post-COVID world.^7^ Without change, inequalities in access to hospice care will continue to grow.

Historically, Māori (indigenous people of Aotearoa/New Zealand) have suffered worse health outcomes and had greater difficulties in accessing health services than non-Māori.^8^ Māori are also less able to access culturally safe specialist palliative care services.^9^ COVID-19 and the resulting lockdowns also challenged the health of whānau Māori (Māori families) alongside their social, cultural, and financial well-being.^10,11^ It is recognised that Māori have unique needs with respect to palliative and end-of-life care, which are not always met by current health services.^12^ This inequity was exacerbated during COVID-19.^13,14^

The COVID-19 pandemic brought increased needs and demands in the community setting, but also provided opportunities for new partnerships and ways of working.^6^ Returning to the status quo was not an option. Rather, moving towards a shared vision and purpose, which has the person and their community network at its centre, enables hospices to have a pivotal role and bring about more equitable palliative care delivery. Because of COVID-19, palliative care services had to adapt rapidly and creatively to find new ways of working, revising, and establishing new policies and practices.^15^ However, these strategies were often adopted ad hoc and on short notice without the benefit of stakeholder consultation.^16^

There is a critical need to harness the lessons learned and examine policy and practice changes made during the COVID-19 pandemic to support safe, high-quality provision. A particular priority is optimising services to best use the limited specialist palliative care workforce and to make sure it is available for those who need it most. Specialist palliative care in New Zealand is care provided by health professionals who have undertaken training specific to palliative care, working as part of an expert interdisciplinary team.^17^ Generalist palliative care is care provided as a central component of usual clinical practice by any healthcare professional who is not a member of a specialist palliative care team.^17^ Dunleavy et al.’s^18^ study has described the flexible adaptations made in the wake of COVID-19. However, we need to go further to understand the human motivation underlying these changes and what will potentially sustain them. Motivation is “any internal process that energises, directs, and sustains behaviour”^19^ (pp. 31–32). Drawing on Deci and Ryan’s self-determination theory (SDT),^20^ this study will explore health professional practice changes in the realms of service, inter- and intra-professional relationships, and cultural responses. SDT proposes three basic psychological needs that must be satisfied if individuals are to achieve health and well-being. These are autonomy (volition – a sense that one has choices), relatedness (a sense of belongingness and connectedness with others), and competence (a sense of capacity and effectiveness).^20^ According to SDT, satisfying these three basic psychological needs promotes intrinsic motivation (inherent human drive to flourish) and self-determined extrinsic regulations.^21^ In other words, satisfaction of all three is a predictor of autonomous types of motivation.^21^ Research has demonstrated that extrinsic stressors can lead to an intrinsic motivation for behaviour change.^22^ This process often involves an initial response to external pressures or challenges (e.g. the pandemic), which over time can foster a deeper, internalised motivation.^23,24^ SDT may provide insights into these changes in thinking and behaviour and the maintenance of those changes. The study aimed to explore the motivations underpinning adaptations and innovations in the delivery of palliative care in the wake of COVID-19.

## Methods

### Design

A Transdisciplinary Action Research (TDAR) approach was utilised. Developed from action research, TDAR involves the contributions of researchers from multiple disciplines working with a range of stakeholders and beneficiaries to jointly address social challenges and identify practical solutions to them.^25^ TDAR is appropriate for tackling complex problems such as the needs of people with life-limiting illnesses.^26^ TDAR supports effective collaboration between behavioural researchers, policymakers, frontline public services staff, and communities.^25^ The TDAR guidance recommends three steps: (1) Evidence review and stakeholder consultation, (2) Co-production, and (3) Prototyping.^27^ The current study focused on step 1. Step 1 involves a process of “cooperative inquiry” (research *with* instead of *on* people) with stakeholders.^28^ The goal of this step is to gather multiple perspectives about innovative solutions to care delivery, organisational and national policy issues post-COVID-19 relevant to palliative care and hospice services for people with life-limiting conditions. This study also included a partnership approach, with both Māori and non-Māori researchers.^29^ *Mātauranga Māori* (Māori knowledge) regarding palliative care delivery post-COVID-19 was facilitated by T.M.M. to ensure new adaptations and innovations are developed with and for Māori.

### Population

Purposive sampling was conducted of hospices, related organisations, and hospice-affiliated health professionals from regions according to the number of COVID-19-positive patients per 100,000 inhabitants, focusing specifically on high (>25 cases) at high prevalence areas during the COVID-19 lockdown as identified in earlier research.^6^ Purposive sampling as a non-probability sampling method was selected based on the characteristics of the population and our study aim.^30^ Participants were recruited by J.T. (who had knowledge of the range of regional hospices and community organisations) to include representatives from hospices, community groups affiliated with hospices, non-governmental organisations, and community care providers, including general practitioners and district nurses.

### Sample

The final sample consisted of representatives from hospices, hospitals, and aged care facilities, government offices associated with older adult issues, non-governmental organisations such as Cancer Society NZ, which provide information and patient support services, general practitioners, nurses, and allied health professionals working in the community. Recruitment of Māori participants reflected the commitment to the centrality of whānau, and thus, participant selection was purposive and facilitated by T.M.M. All participants provided written consent. The questionnaire development and completion process was adapted to incorporate a collectivist worldview. Theoretical saturation across participants from differing health sectors was reached after 37 interviews, with no new codes having been developed.^31^

### Inclusion and exclusion criteria

Potential participants included clinical staff and managers from hospice, clinical staff and managers from residential aged care, managers and staff from community groups affiliated with hospice, managers and staff from non-governmental organisations (e.g. The Cancer Society, Dementia New Zealand), and community care providers including general practitioners and district nurses who work with hospice in delivering palliative care. Exclusion criteria included those community health professionals and members of organisations who did not have a working connection with the hospice.

### Questionnaire

The questionnaire was developed from a literature review and findings from the Health Research Council-funded study on hospice palliative care delivery in New Zealand and Scotland during COVID-19 led by R.F.^15^. Content for the questionnaire was guided by our study aim. The topics covered included the participants’ role, their experiences of palliative care delivery, how COVID-19 impacted care delivery, what innovative solutions were developed to deliver palliative care, and whether those innovative solutions were retained. The majority of the questions took an open-ended format and were thus amenable to qualitative analysis.

### Process

This report represents phase I of a two-phase study on innovations in hospice care for persons with palliative care needs. Phase I (January–August 2024) involved a telephone survey of a sample of hospices and organisations associated with hospice and community care providers as listed above. Stakeholders took part in one-to-one or paired questionnaire completion with a research assistant (E.M.) or researcher using Zoom Video Conferencing or via telephone or in-person (dependent on location). Open-ended question completion was selected rather than interviews to accommodate the time constraints of participants. E.M. and R.F. had no previous association with the participants. Survey completions with an average duration of 45 min were recorded with participant written consent and transcribed by a professional transcriptionist who had signed a confidentiality agreement. T.M.M. completed the questionnaire with Māori participants.

### Analysis

The open-ended responses’ analysis utilised both inductive and deductive thematic analysis^32^ and drew on the concepts provided by SDT.^20^ Thematic analysis was selected because it is flexible, providing direction but allowing the process to be shaped and evolve with the data.^32^ Analysis initially employed deductive processes and drew the components of SDT^20^ to inform the coding (in QSR NVivo 11.0: QSR Intl) of a framework and to help identify relevant textual material.^33^ The three constructs, competence, relationships, and autonomy, were initially applied as codes to the text with the intent of identifying meaningful text units. The subsequent analysis was guided by but not confined to these preliminary codes. An inductive exploration of the open-ended text to identify core themes was followed to explain the factors shaping the quantitative results. Open codes included distinct words or phrases, sentences, or paragraphs, which revealed different concepts, issues, and ideas. Similar codes were merged to form subthemes and themes. Where disagreement occurred, R.F. (social science researcher), J.T. (researcher and nurse practitioner), and E.M. (researcher and nurse) discussed the interpretations and came to a consensus. Utilising the expertise of more than one researcher served to increase inter-rater reliability, rigor, quality, and breadth of the analysis.^34^ T.M.M. conducted the analysis and interpretation of interviews with Māori stakeholders. Limited demographic information is associated with quotes to protect the participants’ identities.

## Findings

### Demographic overview

Most participants were female (81%), ranging in age from 32 to 70, with a mean age of 51 years. Nursing is traditionally a female-dominated profession, providing some explanation for the gender disparity.^7^ Participants most often identified as NZ European (40.5%). The three most often reported roles were nurse (27%), allied health professional (e.g. cultural, spiritual, social work, etc.; 21.6%), and hospice manager (18.9%; Table 1).

**Table 1.** Demographic characteristics of participants.

Variable	Frequency	Percent
Gender		
Female	30	81.1
Male	7	18.9
Age (years)		
32–42	9	25.0
43–53	10	27.8
54–64	13	36.1
65+	4	11.1
Ethnicity		
NZ European	15	40.5
Māori	6	16.2
Pacific	3	8.1
Asian/Southeast Asian	8	21.6
European	3	8.1
Other	2	5.4
Role		
Nurse	10	27.0
Allied health professional (e.g. cultural, spiritual advisors, social work, etc.)	8	21.6
Hospice manager	7	18.9
Physician	5	13.5
Aged care manager	4	10.8
Hospice-affiliated organisation manager	3	8.1

### Thematic analysis

**Figure 1.**
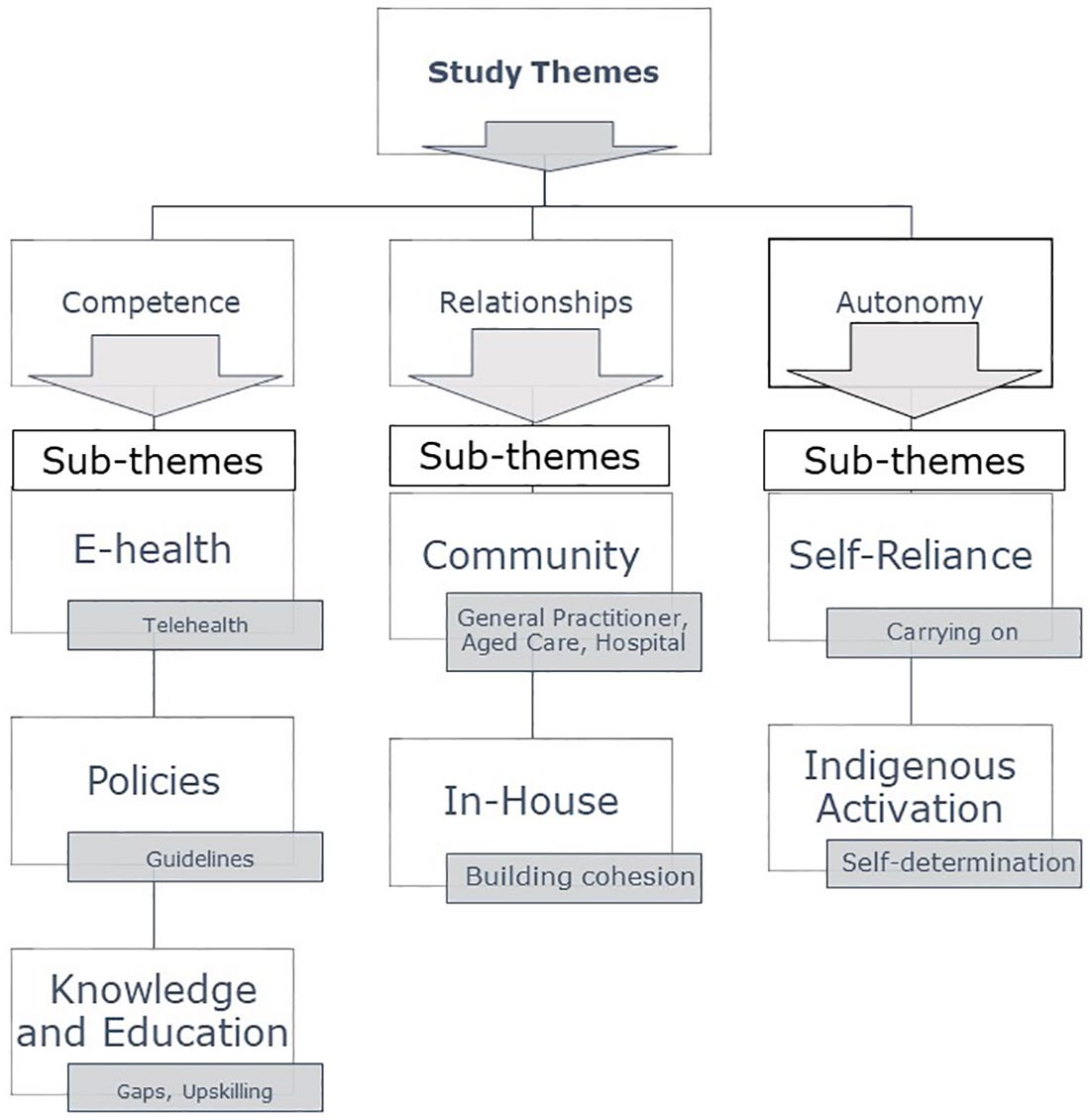
Study themes and subthemes.

The impact of the COVID-19 pandemic has provided an opportunity for healthcare professionals in the end-of-life care sector to rethink strategies, suggestions, and improvements in care for older adults and their family caregivers. Three themes were developed from the open-ended responses and divided into competence, relationships, and autonomy changes. Each motivation theme was broken down into several subthemes related to practice changes, as outlined below. The relationships between themes and subthemes are outlined in Figure 1.

## Competence

In the wake of COVID-19, palliative care services have needed to adapt rapidly and creatively to find new ways of working, revising, and establishing new policies and practices.^16^ These changes provided a stronger sense of competence.

### E-health

Prior to the pandemic, participants reported little engagement with telemedicine. In fact, there was notable resistance to its use. However, health professionals’ rapid uptake and increased familiarity with telemedicine technology overcame this resistance to change.

And most of us were using it [video conferencing] very spasmodically and didn’t have any particular training in it. And so we weren’t pushed to do an IT thing. As soon as we hit COVID, how we needed to be, we needed to be right there. We needed we developed skills very quickly on how to set up meetings. And meet with people online and not, you know, and it was that, you know, look at things coming from Te Whatu Ora [Health New Zealand] online. It was all, you know, little webinar-type things that were coming our way. It was quite, so I think that embedded it, we all knew there was no choice but to do this. So I think it really helped us with our IT sort of world, I think. – (18) General Practitioner

The transition to virtual visits and telemedicine during COVID-19 proved practical for continuing to deliver hospice care and reducing social isolation during restricted visiting. Participants reported that the use of telemedicine has continued.

Still doing. So, some of the virtual stuff, we can still do virtual consults. We can do virtual joint consults. Still, we’re happy to do that. We’re actually sorta digitising some of the tools that we use to look after patients, for example. And so we’re trying to be much more nimble and agile for the next disaster that will come. So, it’s not just okay COVID’s come and there’s some worries about bird flu, but, you know, it’s another crisis. We will be ready for it. We have to sort of be bespoke at the time, but generally, we’ve got a framework going. – (9) Palliative Physician

Forms and policies surrounding telehealth were revised and improved. A hospice manager spoke of the establishment of a formalised process for teleconsultations:

We’ve maintained that. So, we’ve got rid of that initial form. It’s just now a little section on the original consent form that says I, you know, will accept telehealth; this is my preferred method. Yeah, and we’ve maintained that. – (3) Hospice Manager.

The challenges of a lack of patient contact with family were highlighted during the pandemic. In another hospice, changes have been made to increase inpatient access through technology:

I suppose one of the big things, and we’ve changed it now, is ensuring in the room. . .that the TVs were compatible for TEAMS so that the patient could talk to their whānau [family] that are not with them. That has only happened, though, this year. – (31) Community Allied Health

### Guidelines

Guidelines were created and adapted to address the challenges created by COVID-19. These adaptations have been retained and formalised. A palliative care nurse mentioned the revamp of the model of care in response to the pandemic:

I think for us, reviewing our model and not sticking with the status quo was a very courageous thing. It was something that came up from one of our clinical nurse specialists and was a one of those moments of “this might be an unpopular idea, but I'm going to say it anyway”. . . So it was, you know, it was a real courageous thing to do, and it worked for us as a service. – (34) Nurse

### Knowledge and education

Both institutional knowledge and healthcare professional training were updated during and after the pandemic. The goal was to prepare for future emergencies.

### Institutional knowledge

Information developed at speed during the pandemic was centralised for ease of access. There was also an effort to formalise the lessons learned and to make that learning available in the future.

But all the COVID policies, all the information, all the information for volunteers, we’ve now got a massive saved lexicon of everything COVID. So, if anybody who works for [hospice] wants to know anything about what the [hospice] response to COVID is, they click into the COVID-19 folder, and it’s all there. – (3) Hospice Manager

### Training

The pandemic highlighted gaps in clinical skills, particularly within the aged care sector. Nurses in the aged care sector were required to take a larger role in patient assessments than had previously been required. A residential aged care manager highlighted the challenges:

But we had to become their [physicians] hands and eyes and ears and nose. Which was not comfortable for some of the nurses who doesn’t have that assessment skills. . .And a lot of nurses didn’t want to take the responsibility. For example, if the resident complained of cough, and you have to listen to the lungs. And the doctor asks, can you hear any crackles? And the nurse says, what is crackle?. . .What am I supposed to actually be doing? Because they haven’t done that, you know? – (19) Aged Care Manager

However, the pandemic, while highlighting deficiencies, also provided an impetus for new learning opportunities. An aged residential care manager reported on the changes:

We grew in terms of just upskilling and clinical skills, presenting a resident to the nurse practitioner or GP. We’ve done a lot in the way of background and acute assessment. So that when you’re actually in that virtual realm with the nurse practitioner or GP, you’ve actually got a lot of that information there. – (3) Hospice Manager

An aged care facility manager reflected on the growth observed in colleagues, adding:

I don’t think service has changed; I think people changed, I think as health professionals. As nurses, I think we have changed in terms of, you know thinking critically about what was going on. – (17) Aged Care Manager

## Relationships

Relationships with health professionals, patients, and families were altered. These relationships are subdivided into those in the Community and those within the hospice.

### Mending fences

For others, there was a sense of abandonment. These relationships were strained and needed mending. These difficulties were often the result of hospices’ withdrawal of community services during the pandemic.

We’d gotten offside with, because we closed our doors and refused to go and visit people, we’d gotten offside with district nurses, with oncology, with the [general practitioners] GPs. So, it’s taken two years to repair those relationships. – (5) Hospice Manager.

### Growing relationships

COVID-19 led to the establishment and growth of relationships with both aged care and hospital teams. Hospices developed dedicated teams to address the needs of residential care homes. Relationships with hospitals grew stronger as the pandemic increased the need for communication between organisations.

I think our relationship with [hospice nurse] is, you know, it’s taken time. And it is one that we cherish. – (17) Aged Care Manager.

We have a lot to do with X [name of hospital] Hospital and their palliative care team out there. And a bit of contact there. So, our relationship has only grown since with them. And we work very closely together. – (1) Hospice Manager

### In-house relationships

Staff relationships and interactions were altered during the pandemic. For staff, there was an increased sense of collaboration, while for patients and families, there was increased tension and distress.

### Greater cohesion

Lockdowns facilitated “checking in” and sharing of responsibilities rather than siloing of tasks. The activities brought members of the multidisciplinary team closer. It also brought a greater understanding of the roles each plays in the team.

We then started daily Zoom huddles with each team, so we would zoom in, say at nine o’clock in the morning, have these huddles. We don’t Zoom them anymore. . .every base, we have a morning huddle at eight-thirty, and we connect with everybody. We talk about patients that are concerning us. We talk about patients who might be in the hospice inpatient unit or in hospital. And then, as the manager, I go around everyone and go “right, what’s your workload looking like for today?” So, I get a really good idea of how busy each individual member of the team is and where we need to help each other out. – (3) Hospice Manager

### Visitors

The rigid enforcement of restrictions on visitors for hospice and aged residential care facilities profoundly affected patients and families. One hospice manager reported the following assessment:

Caused an enormous amount of distress for patients and families, whether it was the visitor restrictions in our inpatient unit or in the community. There was a lot of distress. – (11) Hospice Manager.

The rigid restrictions and the growing misinformation about the pandemic created tension between families and staff. A residential aged care manager spoke about the challenges posed by anti-vax sentiments prevalent during the latter stages of the pandemic.

I think one of the biggest challenges for us and it was really because we had, you know a 10 to 15 percent of our family population believe the whole thing was a conspiracy. So, and that was just mind-numbing, to be able to manage their behaviour. So, they would drive around, and they would drive around, and they would try and sneak in. – (17) Aged Care Manager

Both hospices and aged care facilities adapted policies to ease the distress. One allied health professional stated that institutions wanted to ensure “*that no one dies alone*” (6), Community Allied Health. In other words, restoring vital connections with families and patients was needed. A residential aged care manager further stated:

We’ve got residents who have COVID now. We nurse them in isolation. However, we don’t restrict visiting. We just work around that to make sure that the visitors are actually safe. That the staff are safe. – (17) Aged Care Manager

## Autonomy

### Self-reliance

The lack of ability to contact hospice led some physicians to rethink care delivery. The result for some community physicians was a new sense of self-reliance, as physicians took on more tasks themselves.

In the sense that I don’t, you know, you’ve learnt a little bit yourself, so you feel like there are some things that you can carry on doing. And it’s only that when I get to the point of I really need their input, that I might reach out to them [Hospice] and contact them. And they have been available when that has happened, but I do feel like I’ve tried to get on and do it myself a bit more. – (15) General Practitioner

### Indigenous activation

Māori responses to COVID-19 drew upon fundamental constructs of *Te Ao Māori* (Māori worldview), which emphasises the collective rather than the individual. More specifically, the flexible, adaptive, and innovative developments described were explicitly embedded within shared and commonly understood cultural values such as *whakapapa* (identity/genealogy), *whānaungatanga* (relationships), *manaakitanga* (support), and *kaitiakitanga* (guardianship). This worldview underpinned a collectivist response that successfully served the Māori community and catalysed a renewed sense of self-determination (*mana motuhake*).

### Wairua

While enhancing physical safety, the personal protective equipment (PPE) caused a barrier between health professionals and patients, leading to an inability to read the wairua (spirit) of the person and establish trust.

I just felt really uncomfortable sitting amongst the whānau. . .I felt there was a barrier with me with all this gear. . .I hated sitting there. And that’s why I felt like when they first came to the door, at least they would have seen me and felt okay. I know what she looked like before she put all the gear on. – (31) Community Allied Health

### Wrap-around care

Whare Tapa Whā is a model of Māori health. Te Whare Tapa Whā uses the wharenui (meeting house) as a symbol for the four cornerstones of Māori health and is based on the concepts of whānau (family), tinana (physical), hinengaro (mental), and wairua (spiritual) health. Evidence of wrap-around care was found in the interviews, including not only delivering health care but also food parcels to persons in the community.

There were some amazing initiatives coming out from [charitable trust], who did a lot of the vaccinations, a lot of door-to-door checking in on people. Delivering food baskets. Just sort of even random cold calling done in some areas. – (15) General Practitioner

Another allied health worker spoke of the importance of Hospice’s reciprocal connections with their Māori community. The local Marae became an integral partner in the delivery of care to the wider community. She stated:

The resources we have here [Hospice] are only enough to support the staff onsite and the patients and whānau [family]. It’s about supporting whānau that are out in the community as well. . .the care packages and the food parcels. . .what would we have done if X marae weren’t available? – (31) Community Allied Health

The pandemic limited access to home-based clinical assistance. Efforts were made to provide further resources for caregivers and families in the care of their family members. This was a shift in thinking meant to empower caregivers as partners in care.

Everything about [ill person care], how to recognise death and dying, because these families have been reliant on clinical teams to give them signals, but now they’re gonna go home. They’re capable, but I think it’s important to prepare them. So having to have those kind of resources, how to recognise what to do, who to go to. So it’s a whole new, different package or conversation. – (10) Hospice Allied Health

### Confidence and capacity

Participants commented on the new level of confidence that had been developed and instilled in whānau as a result of their experiences amid COVID-19. Whānau were quick to implement their increased understanding of the needs of their loved ones. This had the positive effect of an increased sense of empowerment.

Because the whānau (extended family) who had cancer during it [COVID-19 lockdown] and felt like they were empowered to look after their whānau during the palliative care phase. They have said, you know, we wanna carry on doing that. I think the whānau feeling, you know, seeing them so empowered to look after whānau at home is a beautiful thing. I remember one woman going to see her, and she had all her sisters with her, and her kids. And, you know, they would tell me what to do, you know, they’d say, oh yeah, we’ve tried that, we think we need this. And then, you know, go back the next day, oh yeah, that worked perfectly. – (15) General Practitioner

## Discussion

The impact of a worldwide crisis such as the pandemic had a knock-on effect on the organisation of governmental services, with significant consequences for the delivery of health care, including palliative and end-of-life care. In our Aotearoa/New Zealand study, evidence indicates that the resultant adaptations and adjustments to service delivery often continued post-COVID-19. Looking at the SDT model,^20^ motivations for these changes transformed from extrinsic (COVID-19 restrictions) to intrinsic (personally meaningful). That meaning is centred on delivering patient-centred care. These adaptations grew from a shift in thinking about previously established relationships and practices. This shift in thinking and behaviour was illustrated in three areas.

*Competence* – While telehealth has been used for healthcare delivery since the 1950s,^35^ the COVID-19 pandemic accelerated its use. As a result, telehealth has opened up numerous options for patients and physicians in delivering palliative care. In line with Kanavaki et al.’s study,^36^ in this study, digital resources, some of which had been stuck in organisational, cultural, and individual mindsets that were resistant to change, demonstrated rapid uptake by health professionals. Why did this occur? Dunleavy et al.^18^ proposed that extreme crises (e.g. the pandemic) create a “shock” to the established systems such that previously resisted changes are rapidly accepted. The enforced change resulted in a growing sense of competence and comfort with technology.

*Relationships –* While some professionals experienced worsened relationships with hospice, most found that intra-professional collaboration proved effective in adapting to the restrictions posed by the pandemic. Research has indicated that greater inter- and intra-professional collaborations in home-based community care programmes positively impact patient outcomes.^37–39^

Within organisations, lockdowns facilitated “checking in” and sharing of responsibilities rather than siloing of tasks.^40^ These check-ins have continued, although in-person rather than virtually. The effect of these check-ins has been a strengthening of team bonds.^40^ Research in organisational psychology has demonstrated that effective teamwork qualities (e.g. trust, social support, relationship quality among team members, collaborative leadership, and group cohesion) significantly impact team resilience.^41^

Ways of thinking also changed regarding relationships with families and patients. Rigid enforcement of no visitors during COVID-19 led to distress for patients and families. Hospices and aged care facilities responded by adapting policies to ease restrictions. The unpredictability of end-of-life care emphasises the importance of flexible visiting arrangements. In light of pandemic-related restrictions, such flexibility may reduce tension within clinical teams and prevent staff from feeling they have to “break the rules” to deliver optimal family-centred care.^42^

*Autonomy –* Central to the culturally embedded response was a workforce that could facilitate trusting relationships. Similar to research by Moeke-Maxwell et al.,^11^ respondents reported difficulty connecting with patients due to the physical barrier created by PPE. Māori health workers adapted their approach to home visits to address this challenge. Employing cultural values when working with communities was vital. Drawing on relational cultural practices (e.g. *kanohi ki te kanohi – face-to-face*) provides a solid basis for developing the trusting relationships necessary for true engagement.

In line with Cicognani et al.’s^43^ study, the study demonstrated community empowerment, allowing patients and caregivers greater control over the decisions and actions that impact their lives. That feeling of control has remained. Older Mäori (aged 70–79 years) were at greater risk of contracting COVID-19 (a risk of 170.3 per 100,000) among Māori and 264 (82.2 per 100,000) for “European and Other.”^44^ However, overall, Mäori experienced low rates of COVID-19 infection, accounting for approximately 8% of confirmed cases, much below the 16.5% they make up of the national population.^45^ Recognising COVID-19 lockdowns would present a range of issues for whānau, Māori-led responses extended well beyond a narrow focus on physical health as demonstrated in this study and others.^10,46,47^ As illustrated by our study, wrap-around care was provided to the community (e.g. food packages as well as health services). A holistic and relational cultural worldview drove Māori-led responses to the pandemic. Such a worldview prioritises collective well-being, as depicted in Whare Tapa Whā.^48^ A report by McMeeking and Savage^49^ examining Māori responses to COVID-19 stated that pivotal factors in this positive outcome were “Māori mobilisation and self-responsibility.” Russell et al.^47^ identified that Māori responses during COVID-19 created a renewed sense of self-determination (mana motuhake). Indeed, a respondent in our study reported an increased sense of competence and confidence in caregivers as a result of the pandemic. SDT, while formulated within a Western worldview, may be applicable to the shift in thinking and behaviour for persons embedded within a collectivist worldview. Previous research has supported the cross-cultural applicability of SDT^50,51^ and to Māori culture.^52^ The successful outcomes from Indigenous approaches illustrate the vast possibilities able to be realised when Indigenous solutions are enabled. Institutional health services such as hospice can play a role by bringing people’s real needs to the forefront, including them as active agents and key elements in defining solutions that respond to their problems.

### Strengths and limitations

To our knowledge, this study is the first of its kind to provide an in-depth examination of the motivations underlying innovative changes in palliative care delivery in the aftermath of COVID-19. Furthermore, there has been a paucity of research conducted to survey the combined views of multiple stakeholders involved in hospice services and other stakeholders involved in the delivery of palliative care (i.e. partnering with hospice to provide care) because most studies use the experience of care from single perspectives.^53^ Focusing on multiple stakeholders’ experiences and perspectives can provide an evidence-based guide for future planning. It also further facilitates the development of effective relationships in providing integrated palliative care in the Community. Phase II of this study will feature two think-aloud sessions with participants from phase I. The goal will be to produce and or adapt innovative solutions to improve hospice service access and help shape the COVID recovery for palliative care delivery more broadly across the whole country (co-production). It should be noted, however, that as a small, purposive sample, the findings can only be generalised to the (sub)population from which the sample was drawn and not to the entire population.^54^ Following up on this cross-sectional study over time, and with a larger, random sample, will be useful.

## Conclusion

Palliative care services needed to adapt rapidly and creatively to the threats posed by COVID-19. The threats posed were a motivator to shift thinking about palliative care delivery regarding services, relationships, and empowerment. This shift in thinking led to changes in ways of working, revising, and establishing new policies and practices. The driving force underpinning the changes and innovations is the desire to provide patient-centred care. Indeed, the findings build a case for patient-centred, sustainable, and effective innovation. Some of these innovations have the potential to improve access (e.g. telehealth, video conferencing, etc.). From the perspective of health professionals, the findings may inform strategies to sustain new practices in delivering quality palliative care. Additionally, they may also provide insights into possible methods to grow individual and community capacity, including Indigenous communities, to face future pandemics.

## Supplemental Material

sj-docx-1-pcr-10.1177_26323524251343095 – Supplemental material for “I don’t think service changed, I think people changed”: Palliative care delivery in Aotearoa/New Zealand after COVID-19 Supplemental material, sj-docx-1-pcr-10.1177_26323524251343095 for “I don’t think service changed, I think people changed”: Palliative care delivery in Aotearoa/New Zealand after COVID-19 by Rosemary Frey, Tess Moeke-Maxwell, Jackie Robinson, Deborah Raphael, Lisa Williams, Erica Munro, Jenny Thurston and Merryn Gott in Palliative Care and Social Practice

## References

[1] World Health Organization (WHO). Integrated care models: an overview. Geneva: WHO, 2016.

[2] World Health Organisation (WHO). Coronavirus disease (COVID-19) pandemic. Geneva: WHO, 2023.

[3] World Health Organisation. WHO Coronavirus Disease (COVID-19) dashboard. Coronavirus disease (COVID-2019) situation reports. Zurich: WHO, 2022.

[4] Collier A Balmer D Gilder E , et al. Patient safety and hospital visiting at the end of life during COVID-19 restrictions in Aotearoa New Zealand: a qualitative study. BMJ Qual Saf 2023; 32: 704–711. 10.1136/bmjqs-2022-015471 36788035

[5] Dew R Heath L Egan R. Narratives of loss: the impact of COVID-19 lockdown on experiences of loss, grief, and bereavement. J Prim Health Care 2022; 14: 345–351. 36592771 10.1071/HC22090

[6] Frey R Balmer D. COVID-19 and hospice community palliative care in New Zealand: a qualitative study. Health Soc Care Community 2022; 30: e4165–e4174. 10.1111/hsc.13810 PMC9111688 35403763

[7] Sallnow L Smith R Ahmedzai S , et al. Report of the Lancet Commission on the Value of Death: bringing death back into life. Lancet 2022; 399: 837–884. 35114146 10.1016/S0140-6736(21)02314-X PMC8803389

[8] New Zealand. Parliament, Maori Affairs Committee. Pakirehua e pā ana ki ngātaumahatanga hauora mō Ngāi Māori: pūrongo a te Komiti Whiriwhiri Take Māori; Inquiry into health inequalities for Māori: report of the Māori Affairs Committee. Wellington: New Zealand House of Representatives, 2020.

[9] Mason K Black S Moeke-Maxwell T , et al. Maori experiences of palliative care. In: Gott M Robinson J Black S (eds) The voices of underserved communities in palliative care. Wellington, NZ: Te Whatu Ora, 2024, pp. 53–58.

[10] Cassim S Keelan TJ. A review of localised Māori community responses to COVID-19 lockdowns in Aotearoa New Zealand. AlterNative 2023; 19: 42–50. 36967812 10.1177/11771801221124428 PMC10028440

[11] Moeke-Maxwell T Robinson J Gott M. Pōuritanga: Whānau Māori experiences of end-of-life caregiving, death and tangihanga (funeral customs) during New Zealand’s COVID-19 lockdowns. J R Soc N Z 2025; 55: 287–301. 39677382 10.1080/03036758.2024.2345314 PMC11639056

[12] Frey R Gott M Raphael D , et al. “Where do I go from here”? A cultural perspective on challenges to the use of hospice services. Health Soc Care Community 2013; 21: 519–529. 23638970 10.1111/hsc.12038

[13] Megget K. How New Zealand’s covid-19 strategy failed Māori people. BMJ 2022; 376: o224. 10.1136/bmj.o224 35082115

[14] Moeke-Maxwell T Egan R Earp R , et al. Māori spiritual care during COVID-19 lockdowns. In: Best MC (ed.) Spiritual care in palliative care: what it is and why it matters. Springer, 2024, pp. 81–93.

[15] Frey R Balmer D. The challenges for health professionals delivering palliative care in the community during the COVID-19 pandemic: an integrative review. Palliat Support Care 2024; 22: 827–839. 36971027 10.1017/S1478951523000275

[16] Mackwood M Butcher R Vaclavik D , et al. Adoption of telemedicine in a rural US cancer center amid the COVID-19 pandemic: qualitative study. JMIR Cancer 2022; 8: e33768. 10.2196/33768 PMC9384858 35895904

[17] Ministry of Health (NZ). Palliative care action plan. Wellington, NZ: MOH, 2017.

[18] Dunleavy L Preston N Bajwah S , et al. “Necessity is the mother of invention”: specialist palliative care service innovation and practice change in response to COVID-19. Results from a multinational survey (CovPall). Palliat Med 2021; 35: 814–829. 33754892 10.1177/02692163211000660 PMC8114457

[19] Reeve JM. A grand theory of motivation: why not? Motiv Emot 2016; 40: 31–35.

[20] Deci E. Ryan R. Intrinsic motivation and self-determination in human behavior. New York: Plenum, 1985.

[21] Ryan R Deci EL . Self-determination theory: basic psychological needs in motivation, development, and wellness. New York: Guilford Press, 2017.

[22] Fischer C Malycha CP Schafmann E. The influence of intrinsic motivation and synergistic extrinsic motivators on creativity and innovation. Front Psychol 2019; 10: 137. 30778313 10.3389/fpsyg.2019.00137 PMC6369195

[23] Morgado P Cerqueira J. The impact of stress on cognition and motivation. Front Behav Neurosci 2018; 12: 326. 30622462 10.3389/fnbeh.2018.00326 PMC6308156

[24] Morris LS Grehl MM Rutter SB , et al. On what motivates us: a detailed review of intrinsic *v*. extrinsic motivation. Psychol Med 2022; 52: 1801–1816. 35796023 10.1017/S0033291722001611 PMC9340849

[25] Stokols D. Toward a science of transdisciplinary action research. Am J Community Psychol 2006; 38: 63–77. 16791514 10.1007/s10464-006-9060-5

[26] Pineo H Turnbull E Davies M , et al. A new transdisciplinary research model to investigate and improve the health of the public. Health Promot Int 2021; 36: 481–492. 33450013 10.1093/heapro/daaa125 PMC8049543

[27] Hawkins J Madden K Fletcher A , et al. Development of a framework for the co-production and prototyping of public health interventions. BMC Public Health 2017; 17: 689. 28870192 10.1186/s12889-017-4695-8 PMC5583990

[28] Riley S Reason P. Cooperative inquiry. In: Smith JA (ed.) Qualitative psychology: a practical guide to research methods. London: Sage, 2003, pp. 205–231.

[29] Hudson M Milne M Russell K , et al. The development of guidelines for indigenous research ethics in Aotearoa/New Zealand. In: Drugge A-L (ed.) Ethics in Indigenous research, past experiences – future challenges. Umea, Sweden: Vaartoe Centre for Sami Research, Umea University, 2016, pp 157–174.

[30] Alvi M. A manual for selecting sampling techniques in research. Munich: Munich Personal RePEc Archive, 2016.

[31] Dey I. Grounding categories. In: Bryan A Charmaz K (ed.) The SAGE handbook of grounded theory. Thousand Oaks, CA: Sage, 1999, pp. 249–269.

[32] Fereday J Muir-Cochrane E. Demonstrating rigor using thematic analysis: a hybrid approach of inductive and deductive coding and theme development. Int J Qual Methods 2006; 5: 80–92.

[33] Elo S Kyngäs H. The qualitative content analysis process. J Adv Nurs 2008; 62: 107–115. 18352969 10.1111/j.1365-2648.2007.04569.x

[34] Curry LA Nembhard IM Bradley EH. Qualitative and mixed methods provide unique contributions to outcomes research. Circulation 2009; 119: 1442–1452. 19289649 10.1161/CIRCULATIONAHA.107.742775

[35] Frehse A . Overview and history of telehealth. In: Ford DW and Valenta SR (eds) Telemedicine: overview and application in pulmonary, critical care, and sleep medicine. 2021, pp. 3–14.

[36] Kanavaki AM Lightfoot CJ Palmer J , et al. Kidney care during COVID-19 in the UK: perspectives of healthcare professionals on impacts on care quality and staff well-being. Int J Environ Res Public Health 2021; 19: 188. 35010447 10.3390/ijerph19010188 PMC8750502

[37] Manojlovich M Kerr M Davies B , et al. Achieving a climate for patient safety by focusing on relationships. Int J Qual Health Care 2014; 26: 579–584. 25061085 10.1093/intqhc/mzu068

[bibr38-26323524251343095] Mitchell S Harrison M Oliver P , et al. Service change and innovation in community end-of-life care during the COVID-19 pandemic: qualitative analysis of a nationwide primary care survey. Palliat Med 2022; 36: 161–170. 34915759 10.1177/02692163211049311 PMC8796165

[39] van Vuuren J Thomas B Agarwal G , et al. Reshaping healthcare delivery for elderly patients: the role of community paramedicine; a systematic review. BMC Health Serv Res 2021; 21: 1–15. 33407406 10.1186/s12913-020-06037-0 PMC7789625

[40] Abuzied Y Al-Amer R Somduth S , et al. Psychological responses among healthcare workers providing care for patients with COVID-19: a web-based cross-sectional survey in Riyadh, Saudi Arabia. Glob J Qual Saf Healthc 2021; 4: 131–134. 37261222 10.36401/JQSH-21-1 PMC10229035

[41] Morgan P Fletcher D Sarkar M. Defining and characterizing team resilience in elite sport. Psychol Sport Exerc 2013; 14: 549–559.

[42] Hanna J Rapa E Dalton L , et al. Health and social care professionals’ experiences of providing end-of-life care during the COVID-19 pandemic: a qualitative study. Palliat Med 2021; 35: 1249–1257. 34006159 10.1177/02692163211017808 PMC8137863

[43] Cicognani E Albanesi C Valletta L , et al. Quality of collaboration within health promotion partnerships: Impact on sense of community, empowerment, and perceived projects’ outcomes. J Commun Psychol 2020; 48: 323–336. 10.1002/jcop.22254 31596969

[44] Ministry of Health (NZ). COVID-19 mortality in Aotearoa New Zealand: inequities in risk. Wellington, NZ: MOH NZ, 2022.

[45] Ministry of Health (NZ). COVID-19: current cases. Wellington: MOH, 2020.

[46] Te One A Clifford C . Tino Rangatiratanga and well-being: Māori self-determination in the face of COVID-19. Front Sociol 2021; 6: 613340. 33869564 10.3389/fsoc.2021.613340 PMC8022796

[47] Russell L Levy M Barnao E , et al. Enacting Mana Māori Motuhake during COVID-19 in Aotearoa (New Zealand): “We Weren’t Waiting to Be Told What to do.” Int J Environ Res Public Health 2023; 20: 5581. 37107863 10.3390/ijerph20085581 PMC10138436

[48] Durie M. A Maori perspective on health. Soc Sci Med 1985; 20: 483–486. 3992288 10.1016/0277-9536(85)90363-6

[49] McMeeking S Savage C. Maori responses to COVID-19. Policy Q 2020; 16.

[50] Nalipay M King R Cai Y. Autonomy is equally important across East and West: testing the cross-cultural universality of self-determination theory. J Adolesc 2020; 78: 67–72. 31846888 10.1016/j.adolescence.2019.12.009

[51] Church AT Katigbak M Locke K , et al. Need satisfaction and well-being: testing self-determination theory in eight cultures. J Cross Cult Psychol 2013; 44: 507–534.

[52] Ann Roche M Haar JM Brougham D . Māori leaders’ well-being: a self-determination perspective. Leadership 2018; 14: 25–39.

[53] Hiatt K Stelle C Mulsow M , et al. The importance of perspective: evaluation of hospice care from multiple stakeholders. Am J Hosp Palliat Med 2007; 24: 376–382. 10.1177/1049909107300760 17601833

[54] Andrade C. The inconvenient truth about convenience and purposive samples. Indian J Psychol Med 2021; 43: 86–88. 34349313 10.1177/0253717620977000 PMC8295573

