# Supplementary material for: “I don’t think service changed, I think people changed”: Palliative care delivery in Aotearoa/New Zealand after COVID-19
Source: Palliat Care Soc Pract. 2025 Jun 24;19:26323524251343095. doi: 10.1177/26323524251343095 (PMC12188070; doi:10.1177/26323524251343095)
Supplement: sj-docx-1-pcr-10.1177_26323524251343095 – Supplemental material for “I don’t think service changed, I think people changed”: Palliative care delivery in Aotearoa/New Zealand after COVID-19 [file sj-docx-1-pcr-10.1177_26323524251343095.docx]

SRQR Reporting checklist for qualitative study.

**Instructions** **to** **authors**

Complete this checklist by entering the page numbers from your manuscript where readers will find each of the items listed below. SRQR reporting guidelines: O'Brien BC, Harris IB, Beckman TJ, Reed DA, Cook DA. Standards for reporting qualitative research: a synthesis of recommendations. Acad Med. 2014;89(9):1245-1251.

No. Topic Reporting Item Page no.

|  | **Title** **and** **abstract** |  | |
| --- | --- | --- | --- |
| S1 | Title | Concise description of the nature and topic of the study Identifying i | |
|  |  | the study as qualitative or indicating the approach (e.g., | |
|  |  | ethnography, | |
|  |  | grounded theory) or data collection methods (e.g., interview, focus | |
|  |  | group) is recommended | |
| S2 | Abstract | Summary of key elements of the study using the abstract format of ii | |
|  |  | the intended publication; typically includes background, purpose, |  |
|  |  | methods, results, and conclusions |  |
|  | **Introduction** |  |  |
| S3 | Problem formulation | Description and significance of the problem/phenomenon studied;  review of relevant theory and empirical work; problem statement | 1 |
| S4 | Purpose of research or question | Purpose of the study and specific objectives or questions | 2 |
|  | **Methods** |  |  |
| S5 | Qualitative approach and research | Qualitative approach (e.g., ethnography, grounded theory, case study, | 2 |
|  | paradigm | phenomenology, narrative research) and guiding theory if |  |
|  |  | appropriate; |  |
|  |  | identifying the research paradigm (e.g., postpositivist, constructivist/ |  |
|  |  | interpretivist) is also recommended; rationale |  |
| S6 | Research  characteristics and | Researchers’ characteristics that may influence the research,  including | 3 |
|  | reflexivity | personal attributes, qualifications/experience, relationship with |  |
|  |  | participants, assumptions, and/or presuppositions; potential or |  |
|  |  | actual |  |
|  |  | interaction between researchers’ characteristics and the research |  |
|  |  | questions, approach, methods, results, and/or transferability |  |
| S7 | Context | Setting/site and salient contextual factors; rationale | 1-2 |
| S8 | Sampling strategy | How and why research participants, documents, or events were | 3-4 |
|  |  | selected; criteria for deciding when no further sampling was |  |
|  |  | necessary |  |
|  |  | (e.g., sampling saturation); rationale |  |
| S9 | Ethical issues | Documentation of approval by an appropriate ethics review board | 3 |
|  | pertaining to human | and participant consent, or explanation for lack thereof; other |  |
|  | subjects | confidentiality and data security issues |  |
| S10 | Data collection  methods | Types of data collected; details of data collection procedures  including | 2-3 |
|  |  | (as appropriate) start and stop dates of data collection and analysis, |  |
|  |  | iterative process, triangulation of sources/methods, and |  |
|  |  | modification |  |
|  |  | of procedures in response to evolving study findings; rationale |  |
| S11 | Data collection  instruments and | Description of instruments (e.g., interview guides, questionnaires)  and devices (e.g., audio recorders) used for data collection; if/how | 2 |
|  | technologies | the |  |
|  |  | instrument(s) changed over the course of the study |  |
| S12 | Units of study | Number and relevant characteristics of participants, documents, or events included in the study; level of participation (could be | 2 |
|  |  | reported |  |
|  |  | in results) |  |
| S13 | Data processing | Methods for processing data prior to and during analysis, including | 3 |

|  |  | transcription, data entry, data management and security, verification  of data integrity, data coding, and anonymization/deidentification of excerpts |  |
| --- | --- | --- | --- |
| S14 | Data analysis | Process by which inferences, themes, etc., were identified and | 3 |
|  |  | developed, including the researchers involved in data analysis; |  |
|  |  | usually |  |
|  |  | references a specific paradigm or approach; rationale |  |
| S15 | Techniques to | Techniques to enhance trustworthiness and credibility of data | 3 |
|  | enhance | analysis |  |
|  | trustworthiness | (e.g., member checking, audit trail, triangulation); rationale |  |
|  | **Results/Findings** |  |  |
| S16 | Synthesis and | Main findings (e.g., interpretations, inferences, and themes); might | 3-8 |
|  | interpretation | include development of a theory or model, or integration with prior |  |
|  |  | research or theory |  |
| S17 | Links to empirical data | Evidence (e.g., quotes, field notes, text excerpts, photographs) to | 3-8 |
|  |  | substantiate analytic findings |  |
|  | **Discussion** |  |  |
| S18 | Integration with prior  work, implications, | Short summary of main findings; explanation of how findings  and conclusions connect to, support, elaborate on, or challenge | 8-9 |
|  | transferability, and | conclusions of earlier scholarship; discussion of scope of application/ |  |
|  | contribution(s) to the | generalizability; identification of unique contribution(s) to |  |
|  | field | scholarship |  |
|  |  | in a discipline or field |  |
| S19 | Limitations | Trustworthiness and limitations of findings | 9 |
|  | **Other** |  |  |
| S20 | Conflicts of interest | Potential sources of influence or perceived influence on study | 10 |
|  |  | conduct |  |
|  |  | and conclusions; how these were managed |  |
| S21 | Funding | Sources of funding and other support; role of funders in data | 10 |
|  |  | collection, interpretation, and reporting |  |

The SRQR checklist is distributed with permission of Wolters Kluwer © 2014 by the Association of American Medical Colleges.

This checklist can be completed online using https://[www.goodreports.org/,](http://www.goodreports.org/) a tool made by the EQUATOR Network in collaboration with Penelope.ai
